# Role of MXD3 in Proliferation of DAOY Human Medulloblastoma Cells

**DOI:** 10.1371/journal.pone.0038508

**Published:** 2012-07-10

**Authors:** Gustavo A. Barisone, Tin Ngo, Martin Tran, Daniel Cortes, Mehdi H. Shahi, Tuong-Vi Nguyen, Daniel Perez-Lanza, Wanna Matayasuwan, Elva Díaz

**Affiliations:** Department of Pharmacology, University of California Davis School of Medicine, Davis, California, United States of America; National Cancer Center, Japan

## Abstract

A subset of medulloblastomas, the most common brain tumor in children, is hypothesized to originate from granule neuron precursors (GNPs) in which the sonic hedgehog (SHH) pathway is over-activated. *MXD3*, a basic helix-look-helix zipper transcription factor of the MAD family, has been reported to be upregulated during postnatal cerebellar development and to promote GNP proliferation and MYCN expression. *Mxd3* is upregulated in mouse models of medulloblastoma as well as in human medulloblastomas. Therefore, we hypothesize that MXD3 plays a role in the cellular events that lead to medulloblastoma biogenesis. In agreement with its proliferative role in GNPs, *MXD3* knock-down in DAOY cells resulted in decreased proliferation. Sustained overexpression of MXD3 resulted in decreased cell numbers due to increased apoptosis and cell cycle arrest. Structure-function analysis revealed that the Sin3 interacting domain, the basic domain, and binding to E-boxes are essential for this activity. Microarray-based expression analysis indicated up-regulation of 84 genes and down-regulation of 47 genes. Potential direct MXD3 target genes were identified by ChIP-chip. Our results suggest that MXD3 is necessary for DAOY medulloblastoma cell proliferation. However, increased level and/or duration of MXD3 expression ultimately reduces cell numbers via increased cell death and cell cycle arrest.

## Introduction

Tumors of the central nervous system (CNS) comprise nearly one quarter of all pediatric cancers. Among them, medulloblastomas, embryonic neuroepithelial tumors of the cerebellum, are the most common [1]. Subsets of medulloblastomas are thought to have different developmental origins. “SHH-type” medulloblastomas originate from a distinct population of cells within the cerebellum - granule neuron precursors (GNPs) - in which the SHH pathway is persistently activated [2]. During normal development, GNPs proliferate in response to SHH [3], and later differentiate and migrate to the internal granule layer. Two of the most dramatically SHH-induced genes are *Dcyclin* and *Mycn* [4]. MYCN is a member of the MYC/MAX/MAD network of the basic helix-loop-helix leucine zipper (bHLHZ) DNA binding proteins. MYC and MAD form heterodimers with the cofactor MAX to achieve binding to the E-box sequence CANNTG [5]. It is accepted that MYC/MAX heterodimers promote proliferation by activating transcription of cell cycle-specific genes through mechanisms that include recruitment of histone acetyl-transferase (HAT) activity [6]. In contrast, MAD/MAX heterodimers repress transcription by recruiting histone deacetylase (HDAC) activity [7], thereby antagonizing MYC/MAX heterodimers, leading to proliferation arrest and differentiation. Hence, current models suggest that the MYC/MAX/MAD network plays an important role in the switch from proliferation (MYC/MAX) to differentiation (MAD/MAX) [5].

The Mad family of transcription factors consists of four paralogous genes: *MXD1*, *MXI1*, *MXD3* and *MXD4*. Consistent with the proliferation/differentiation switch paradigm, genetic ablation of *mxd1* in mice results in defects in cell cycle exit during myeloid differentiation [8], and disruption of the *mxi1* locus results in hyperplasia in several tissues [9]. Several reports, however, suggest that MXD3 is an atypical member of the MAD family. Mice with a targeted deletion of *Mxd3* showed a mild phenotype consisting of increased sensitivity to apoptosis in response to DNA damage [10]. In the developing mouse embryo, *Mxd1*, *Mxi1* and *Mxd4* are expressed in postmitotic cells while *Mxd3*, in contrast, is only present in mitotic cells during the S-phase of the cell cycle [11]–[13]. *Mxd3* was identified as being upregulated during cerebellar GNP development [14], and in a previous study from our lab, MXD3 was shown to be expressed in response to SHH stimulation, and to be necessary and sufficient for cerebellar GNP proliferation [15], challenging the current paradigm that Mad proteins arrest proliferation and promote differentiation by antagonizing Myc function. In agreement with this challenge, *Mxd3* has recently been reported to be upregulated in immature B cells in mouse spleen, where it negatively regulates B cell differentiation [16]. Furthermore, MXD3 is expressed in tumors derived from *patched* heterozygous (*ptc^+/−^*) mice (a model for medulloblastoma, [17]) but not in surrounding normal mature cerebellar tissue [15]. Finally, MXD3 appears to be expressed in human medulloblastomas [18], suggesting a role in medulloblastoma biogenesis, maintenance and/or proliferation.

To test this hypothesis, we studied the role of MXD3 in medulloblastoma using the human medulloblastoma cell line DAOY. Here, we present evidence that MXD3 promotes proliferation; however, sustained high levels of the protein negatively influence proliferation of medulloblastoma cells by the induction of programmed cell death. We report patterns of gene expression in MXD3-overexpressing cells, as well as candidate direct target genes, as a first effort to elucidate mechanisms of MXD3 function and its role in tumor cell proliferation and tumorigenesis pathways.

## Results

### MXD3 is Expressed in Human Medulloblastomas

**Figure 1:**
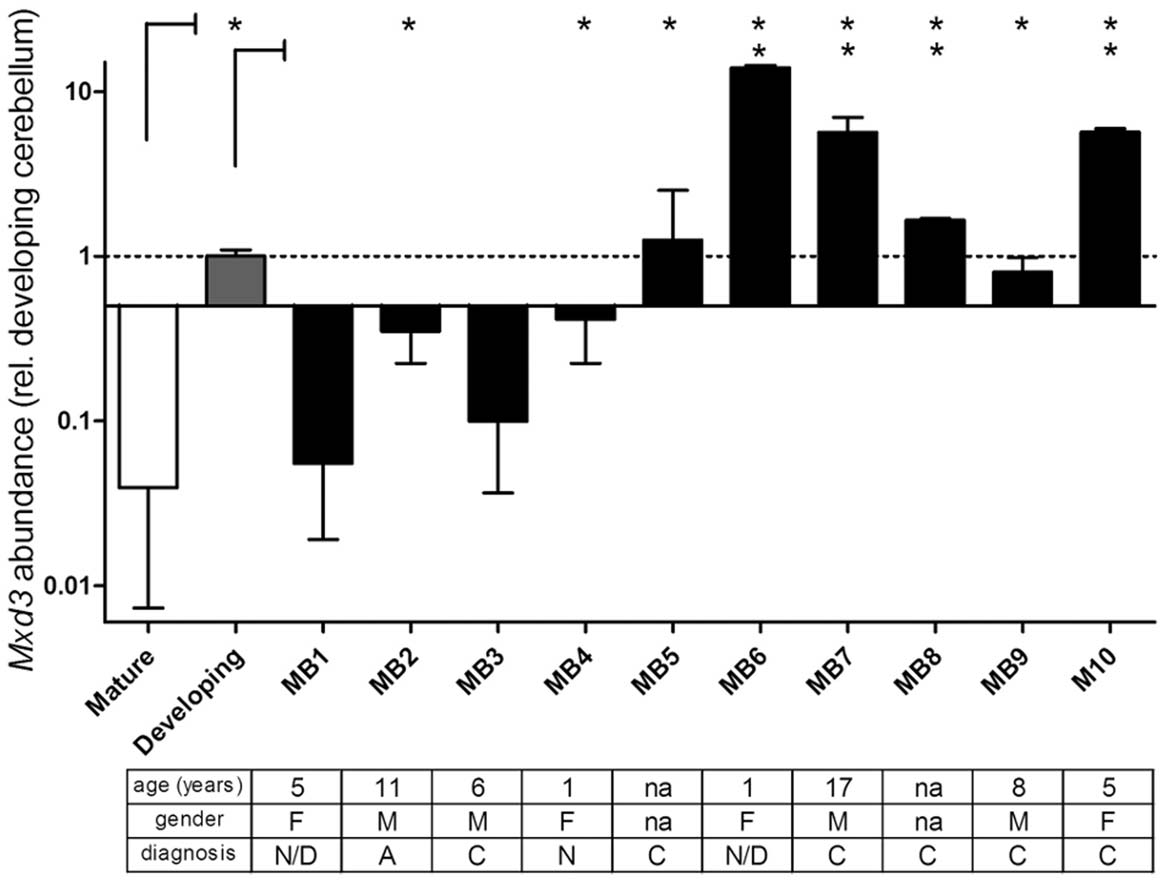
Expression of *MXD3* in human medulloblastomas. *MXD3* mRNA levels in ten human medulloblastoma samples, normal developing and normal mature cerebellum determined by quantitative RT-PCR analysis. Values represent the mean fold-difference in mRNA (n  = 4) relative to developing cerebellum. Error bars indicate standard deviation (SD). Note the logarithmic scale of the Y-axis. The developing cerebellum sample and eight out of ten medulloblastomas showed significantly higher expression of *MXD3* when compared to normal mature tissue (asterisks, *p*<0.01; one-way ANOVA).

Since MXD3 promotes GNP proliferation during normal cerebellar development and it is abnormally expressed in cerebellar tumors in *ptc^+/−^* mice [15], we reasoned that it might play an important role in the pathways that lead to uncontrolled proliferation in human medulloblastoma. Indeed, analysis of expression databases suggested that MXD3 is expressed in many human neoplasias, and in particular in tumors of the CNS, most significantly in glioblastomas and medulloblastomas [18], while it is absent in most human adult tissues. MXD3 is expressed in normal cerebellum during the GNP expansion. GNPs cease to proliferate shortly after birth and, during the first 2 years of life in humans, they differentiate as they migrate to form the internal granular layer (IGL). Accordingly, we observed very low levels of MXD3 in mature cerebellum. As shown in **Fig. 1**, MXD3 levels in mature cerebellum (where granular neurons are not proliferating) is 2 orders of magnitude lower than in developing cerebellum (where GNPs are proliferating). Interestingly, 8 out of 10 human medulloblastoma samples analyzed showed *MXD3* levels significantly higher than normal mature cerebellum (p<0.05). Matched normal tissue was not available for analysis; nonetheless, since patient ages ranged from infants to adolescents (Fig. 1), MXD3 levels in normal tissue is expected to be comparable to a mature cerebellum sample. Even more, 4 of the tumors showed levels of MXD3 significantly higher than those observed in fetal developing cerebellum. Taken together, these results indicate that abnormally high MXD3 expression is a characteristic of at least a subset of medulloblastomas.

To validate our experimental model used for the rest of this study, MXD3 expression in the DAOY cell line was analyzed. DAOY was chosen as it is the only medulloblastoma-derived cell line that can be grown in a monolayer, passed as single-cell suspension and easily transfected; these represent obvious technical advantages for cell counting, immunocytochemistry, over-expression and knock-down experiments. *MXD3* cDNA was cloned from DAOY total RNA extracts; the full coding sequence obtained was 100% identical to the wild-type sequence (NM_031300). Immunoblot analysis failed to show any specific band using three different commercial anti-MXD3 antibodies. These results suggest that although the wild-type message is present, MXD3 protein may be expressed at very low levels, below the limit of detection for the antibodies available. Indeed, analysis by qRT-PCR showed that *MXD3* transcript is present in DAOY cells at low levels (**Fig. S1**).

### MXD3 in DAOY Cell Proliferation

**Figure 2:**
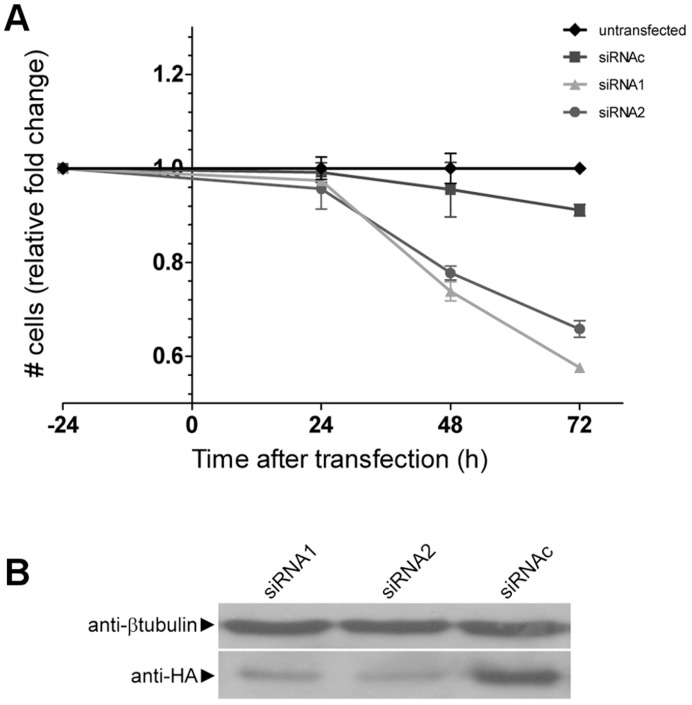
MXD3 knock-down decreases DAOY medulloblastoma cell proliferation. (**A**) Loss of MXD3 decreases DAOY cell proliferation. Quantification of cell proliferation (as total number of cells relative to control) of DAOY cells transfected with *MXD3* (siRNA1, 2) or control (siRNAc) siRNAs. Knock-down of endogenous MXD3 resulted in reduced DAOY cell counts (asterisks, *p*<0.05; n  = 3; two-way ANOVA). (**B**) siRNA-mediated reduction of HA-MXD3 expression. DAOY cells were transiently co-transfected with HA-*MXD3* and siRNAs as in (A). Extracts were probed with anti-HA antibody to detect HA-MXD3 (lower panel) or anti-tubulin antibodies for load control (upper panel).

To assess MXD3’s role in the proliferation of DAOY cells, we performed knock-down and overexpression experiments. Knocking down the endogenous protein with two different specific siRNAs in DAOY cells resulted in a significant decrease in proliferation: total cell numbers were reduced to 75–80% after 48 hrs and 65–55% after 72 hrs when compared to untransfected cells (**Fig. 2A**). In comparison, transfection of control siRNA (siRNAc) resulted in only 5–10% reduction, which is attributed to toxicity of the procedure. Thus, reduced levels of MXD3 result in reduced proliferation in this cell culture system. Knockdown efficiency was validated in DAOY cells cotransfected with HA-tagged MXD3 (HA-MXD3), since the endogenous protein cannot be detected in immunoblots with available antibodies. Both siRNAs reduced HA-MXD3 protein expression by at least 60% (**Fig. 2B**; siRNA1, 68±5%; siRNA2, 90±10%) when compared to control siRNAc transfected cells. These data imply that MXD3 is necessary for medulloblastoma cell proliferation, in agreement with the previously reported effect during proliferation of non-tumor GNPs [15]. Furthermore, although MXD3 protein in DAOY cells could not be detected due to technical limitations of the available antibodies, the confirmation of a wild type mRNA and the observation of a phenotype upon MXD3 knock-down consistent with previous observations in cerebellar GNPs suggests not only the presence but also the biological relevance of this protein in DAOY cell proliferation.

As an experimental tool for genomic approaches to identify MXD3 target genes (see next section), DAOY cells were engineered to stably express HA-MXD3. A number of stable transfectants carrying either the HA-*MXD3* construct or the empty vector were isolated under G418 selection. Several *MXD3* (“M”) and control (“C”) clones were characterized in terms of MXD3 expression. *MXD3* mRNA levels as measured by qRT-PCR were increased 30 to 1,700 times in *MXD3* lines (M1–M5, **Fig. S3A**). Protein levels reflected the magnitude of message levels. Cellular fractionation followed by immunoblot analysis confirmed that, as expected, HA-MXD3 was present exclusively in the nuclear fraction (**Fig. S3B**). Furthermore, immunofluorescence on fixed, permeabilized cells showed HA-MXD3 localized to nuclei (**Fig. S3C**). Therefore, the “M” cell lines express HA-MXD3 in the appropriate subcellular compartment. “C” cell lines showed *MXD3* mRNA levels indistinguishable from the parental cell line.

**Figure 3:**
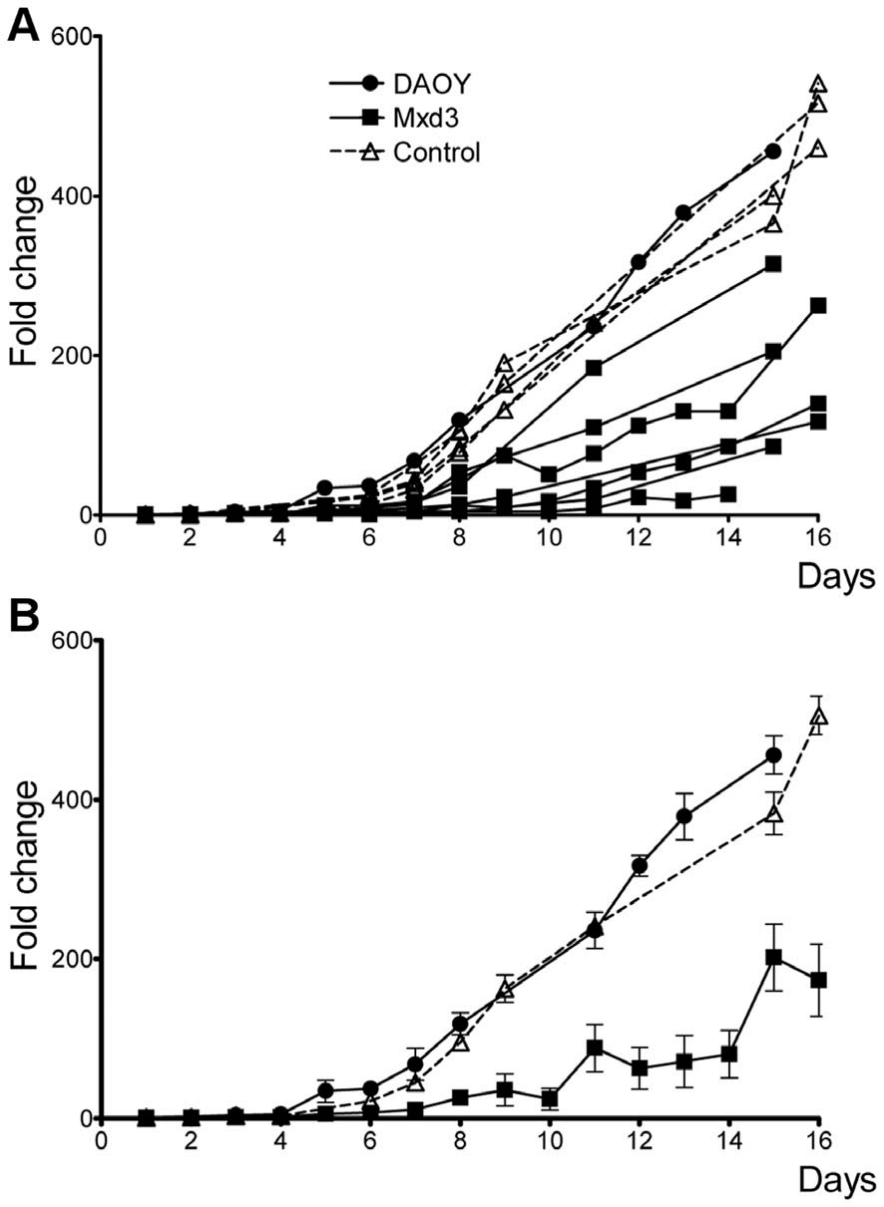
Persistent expression of MXD3 reduces cell proliferation. (**A**) Proliferation curves for DAOY (filled circles), individual “M” (filled squares) and “C” (open triangles) cell lines expressed as fold increase from starting cell count. Each data point represents the mean ± SD of 2–3 independent experiments. All cell lines overexpressing MXD3 showed reduced cell proliferation. Control lines were not different from the parental line. For clarity, the same traces are used for different cell lines within each group. (**B**) For statistical analysis, proliferation data from all cell lines in (A) were pooled into 3 groups: DAOY (filled circles), MXD3 (filled squares) and control (open triangles). From day eight onwards, MXD3 group showed significantly slower proliferation than the parental or control groups (p<0.01, n  = 15, two-way ANOVA with Scheffe post-test for individual comparisons between groups).

Surprisingly, when proliferation rates of the cell lines were analyzed, we observed decreased cell numbers in “M” lines when compared to parental or control lines in proliferation assays. Seven individual “M” clones and four individual “C” clones were analyzed to avoid non-specific phenotypes associated with random DNA insertion events inherent in the generation of stable cell lines (**Fig. 3A**). For statistical analysis, all data were pooled into an “MXD3” group and a “Control” group. As shown in **Fig. 3B**, the MXD3 group had a significantly lower proliferation rate when compared to the control group or the parental (p<0.001). This difference was evident as early as the beginning of the logarithmic proliferation phase (day 7, p<0.05) and reached a maximum towards the end of such phase, before the proliferation plateau. Indeed, on day 15, while “C” lines had a mean fold increase in cell number (relative to day 1) of 392.6, “M” lines showed a mean fold increase of only 225.4, representing a MXD3-mediated decrease of 42% (p<0.003) in cell proliferation.

**Figure 4:**
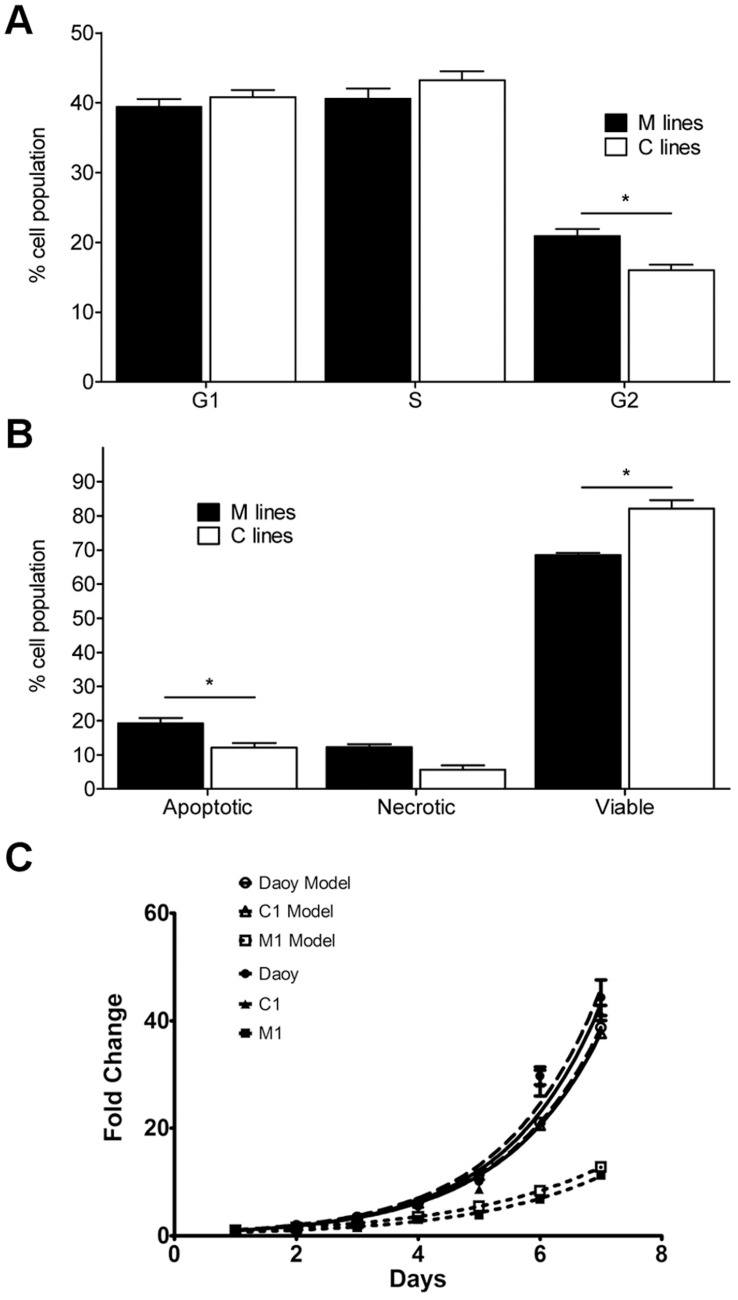
MXD3 influences cell cycle and apoptosis. (**A−B**) Cell cycle (A) and apoptosis (B) analysis by flow cytometry of MXD3 and control cell lines. Bars represent the average ± SD of two independent experiments, each performed on four MXD3 lines (“M”) and four control lines (“C”). MXD3 overexpression resulted in a significantly higher percentage of the population in the G2 phase and increased apoptosis (p<0.05, two-way ANOVA). (**C**) Theoretical proliferation curves (empty symbols) and experimental data (full symbols) for representative cell lines.

To dissect the mechanism underlying this effect, cell cycle progression and apoptosis were studied in “M” and “C” cell lines. Again, to account for individual cell line variations, four of each cell lines were analyzed. As shown in **Fig. 4A**, “M” cell lines presented on average a small but significant increase in the G2 population, with no significant changes in the G1 or S populations. Therefore, persistent MXD3 overexpression seems to result in a longer G2 phase. Staining of the same eight cell lines for apoptosis indicated a significant increase in the apoptotic fraction in “M” lines (**Fig. 4B**). Taken together, these results suggest that sustained high levels of MXD3 ultimately result in cell cycle arrest and trigger apoptosis, thus accounting for the observed reduced total number of cells in our experiments. Indeed, when these two factors were input into a derivative of the exponential growth model (N_(t)_  =  N_0_(1+ r/100)^t/d^), the predicted growth curves showed a very good fit to the experimental data (**Fig. 4C**). Hence, G2 arrest and increased apoptosis seem to account for the experimentally determined difference between the proliferation rates of our “M” and “C” lines.

### MXD3 Structure-function Analysis

**Figure 5:**
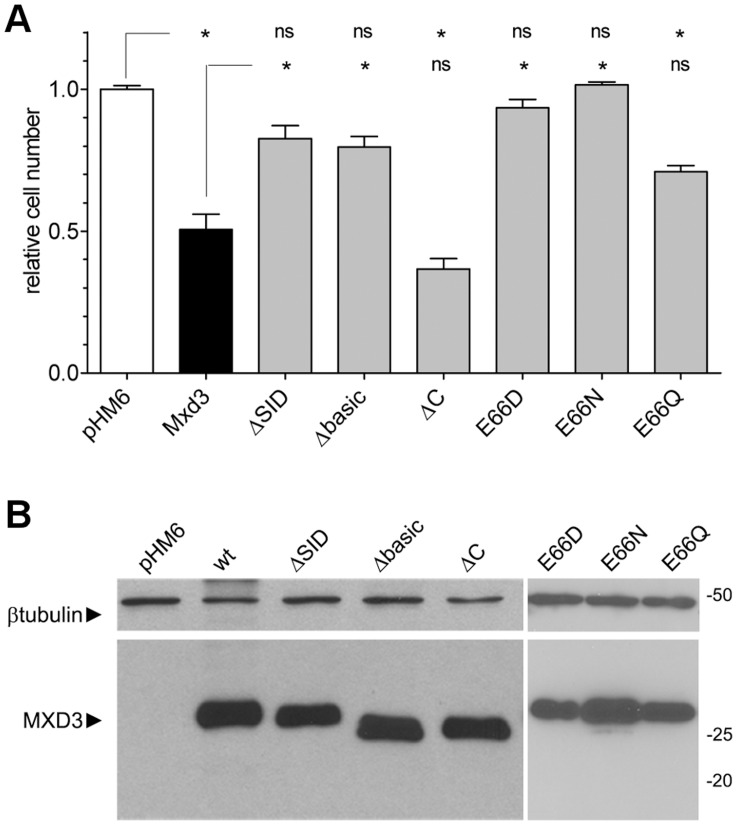
MXD3 structure-function analysis. (**A**) Quantification of cell proliferation (as total number of cells relative to control) of DAOY cells transfected with full-length MXD3 or mutated forms as indicated in Figure S2. MXD3-transfected cells showed 50% reduction of proliferation compared with vector-only (pHM6). Transfection of ΔC and E66Q constructs resulted in similar reduction in proliferation as full-length MXD3. Transfection of the ΔSID, Δbasic, E66D and E66N constructs had no effect on proliferation compared to control (ns, not significant; asterisks, p<0.001; n  = 4, one-way ANOVA with Bonferroni’s multiple comparison test). (**B**) Immunoblot analysis of total protein extracts from DAOY cells transfected with the MXD3 constructs in (B). All constructs used expressed the expected proteins at similar levels. Bottom panel, anti-HA; top panel, anti-β-tubulin used as a load control. Molecular mass markers are indicated on the right.

MXD proteins consist of two functional modules. The bHLHLZ domain mediates heterodimerization with other factors (most importantly, Max) via the second helix and the leucine zipper, and DNA binding through the basic region [19]. The Sin-Interaction Domain (SID [7]) mediates recruitment of a repressor complex via interaction with mSin3 proteins. To dissect the domains required for MXD3 overexpression activity, deletion constructs were generated in which the SID domain (ΔSID) or the basic region of the bHLHZ domain (Δbasic) were removed. As shown in **Figure 5**, overexpression of HA-MXD3 by transient transfection resulted in decreased cell numbers after 48 hrs. Both deletions, however, completely abrogated this phenotype, suggesting that binding to DNA and interaction with, presumably, mSin3 cofactors are essential. Three amino acid residues within the basic region are responsible for direct, specific contact with DNA bases: H62, E66 and R70 [19]–[21] (MXD3 numbering, according to RefSeq accession NP_112590). To investigate further the requirement for DNA binding, the glutamic acid at position 66 was substituted with an aspartic acid residue. Although E to D is a conservative substitution, it has been shown that this substitution completely abolishes DNA binding of the bHLH protein PHO4 [22], [23]. Accordingly, E66D substitution resulted in complete disruption of MXD3 activity in our experimental model (**Fig. 5A**). It has been suggested that the length of the side chain rather than the nature of the functional group is important for the interaction [22]. Accordingly, the E66Q mutation showed no difference in activity (p<0.05) compared to the wild type, while E66N resulted in a complete loss of activity. Importantly, these mutations have been shown in other bHLH proteins to result in binding or no binding, respectively, to DNA target sequences by gel-shift assays [22]. Finally, deletion of the C-terminal 14 amino acid residues of MXD3 (ΔC), a region highly conserved in all MXD proteins, did not have any significant effect (**Fig. 5A**). A schematic representation of the constructs used is presented in **Fig. S2**. Protein expression levels of the constructs used were found to be similar in all cases (**Fig. 5B**), and localization to the nucleus was confirmed (**Fig. S3**).

### MXD3 Direct Transcriptional Targets and Downstream Genes

The results presented thus far suggest a role for MXD3 in medulloblastoma proliferation. The next goal was to shed light into the genetic programs that govern the phenotypes observed. According to the current paradigm, MXD3 is expected to work as a transcriptional repressor by binding promoter sequences as a heterodimer with MAX. However, its role as a transcriptional regulator is mostly inferred from experimental data available for other MXD family members. Furthermore, it is important to note that specific MXD3 gene targets have not yet been identified. We decided to address this question through a genomic approach by performing ChIP-chip on our stable cell lines with high expression of HA-MXD3. Taking advantage of the HA tag fused to the *MXD3* coding sequence, commercial anti-HA antibodies previously used successfully in ChIP experiments [24] were employed. Since no MXD3 target genes have been identified to serve as controls, two types of positive controls were used. First, samples were immunoprecipitated in parallel with anti-RNA polymerase II antibodies. Enrichment of specific targets as confirmed by PCR indicated that the technique worked consistently under our experimental conditions (not shown). Secondly, MC7-E2F1 cells that stably express HA-tagged E2F1 [25] were immunoprecipitated in parallel with the same anti-HA antibody. PCR confirmed enrichment of E2F1 targets in the immunoprecipitate as previously reported [25] (data not shown), thereby validating the use of the anti-HA antibody in our experimental conditions.

**Figure 6:**
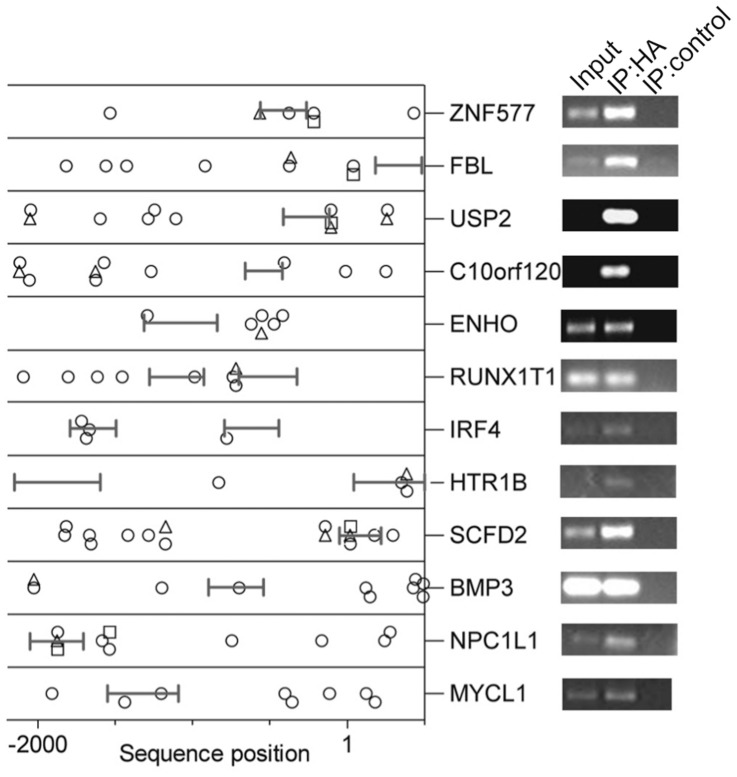
Identity of MXD3 direct target genes. MXD3 direct target genes from ChIP-chip analysis were validated by PCR on an independent ChIP sample. Specific bands are shown in the panels on the right. Transcription factor binding prediction analysis indicated the presence of E-boxes of the c-Myc (○), Max (▵) and N-Myc (□) type. Symbols represent the position of the predicted binding site, relative to the transcription start site. The position of the peak for MXD3 binding, as determined by Maxfour analysis of ChIP data, is indicated by a line (**|**–**|**).

To identify MXD3 binding sites in our “M” cell lines, we performed ChIP for hybridization to DNA microarrays. Differentially labeled immunoprecipitated and input DNA was cohybridized to Nimblegen HG18 RefSeq human promoter arrays. Data were analyzed using NimbleScan software and binding sites were ranked using Maxfour as described [26]. Using permissive criteria (**Table S1**), 788 genes whose promoter sequences were enriched in the immunoprecipitated material compared to the input sample were identified (**Table S1**). A subset of the highest ranking candidates was confirmed by PCR on an independent ChIP sample (12 ChIP hits, **Fig. 6**). Candidates were considered to be confirmed when PCR-amplification showed enrichment in the immunoprecipitated material and were absent in the control (IgG) immunoprecipitate. The list of validated MXD3 targets is presented in **Table 1**. Since members of the MXD family are known to bind E-boxes, we reasoned that these DNA motifs should be present in the promoters of these genes. Indeed, using PROMO [27], [28], E-boxes were identified in all of the genes in **Table 1** (**Fig. 6**). All E-box binding proteins bind the common consensus CANNTG, while target specificity for each transcription factor is thought to depend on the bases flanking that consensus sequence; based on this, algorithms have been defined to identify E-box “subtypes” [27], [28]. Interestingly, MXD3 binding regions (as determined by ChIP-chip) in the promoters of the above genes overlap with predicted MYC binding sites (as suggested by the presence of MYC-type E-boxes, **Fig. 6**) in 11 out of 12 genes analyzed. MAX-type and MYCN-type E-boxes are found in MXD3 binding regions in 6 and 4 of the above genes, respectively. Notably, 6 genes (MYCL1, BMP3, IRF4, RUNX1T1, ENHO and C10orf120) only contain MYC-type E-boxes in MXD3 binding regions.

**Table 1:** Validated MXD3 target genes.

Gene name*	*Accession*	*ChIP 1* ¶	*ChIP 2* ¶	*Expression profile (MXD3/Ctrl)* &		*Biological process (relevant to this study)*
ZNF577	NM_032679	2.9	2.54	1.80	**↑**	unknown function; aberrant methylation and differential gene expression in several malignancies [37]
RUNX1T1	NM_175635	0.2	3.70	2.77	**↑**	senescence-like growth arrest [38]; metastasis [39]
IRF4	NM_002460	1.0	3.78	3.60	**↑**	inhibition of tumor cell death [40]
SCFD2	NM_152540	3.1	3.08	1.39	**↑**	unknown; regulated by tumor suppressor p53 [41]
FBL	NM_001436	3.2	2.78	1.16	**↑**	cell growth in HeLa [42]; overexpression in prostate cancer linked to MYC oncogene [43]
ENHO	NM_198573	3.5	3.63	1.18	**↑**	unknown
NPC1L1	NM_013389	2.0	2.54	2.16	**↑**	unknown
HTR1B	NM_000863	1.7	5.18	1.61	**↑**	T-cell proliferation [44]
BMP3	NM_001201	0.5	3.47	1.83	**↑**	mesenchymal stem cell proliferation [45]; tumor suppression [46]
USP2	NM_004205	2.9	5.11	0.71	**↓**	tumor cell proliferation [47]; tumorigenesis,anti-apoptotic [48]
MYCL1	NM_005376	0.1	3.01	0.95	**↓**	proto-oncogene; amplified in human gliomas [49] and medulloblastomas [50]
C10orf120	NM_001010912	3.1	3.53	n/a		unknown

* The complete list is presented in Table S1.

¶ ChIP 1 and ChIP 2 represent the peak values of 2 independent experiments.

& Values represent fold increase in mRNA abundance calculated from expression profiling arrays as the average normalized signal across MXD3 lines over the average normalized signal across control lines. Arrows indicate upregulation or downregulation.

Next, changes in patterns of gene expression in DAOY cells upon expression of MXD3 were identified. RNA was extracted from four “M” lines, three “C” lines, and the parental DAOY cells, labeled and hybridized to Illumina human expression arrays. Data were pooled into groups for identification of genes that were up-regulated or down-regulated (>2-fold change) in *MXD3* versus control lines. Genes that were differentially expressed in control lines when compared to the parental were excluded from further analysis regardless of the level of expression in *MXD3* lines. Hence, only genes differentially expressed in all “M” lines but in none of the “C” lines compared to the parental were considered. This analysis identified 131 genes that were differentially expressed (**Table S1**). Of those, 47 were down-regulated and 84 were up-regulated.

Genes that were directly bound by MXD3 and showed different levels of expression were submitted to gene ontology analysis using DAVID [29], [30]. The most relevant functional categories included neurogenesis and nervous system development, reflecting previously reported roles of MXD3; interestingly, several categories were related to cancer biology, including apoptosis, cell differentiation and regulation of cell proliferation, suggesting a role for MXD3 in tumorigenesis in support of the hypothesis in this study. By far, most of the genes fell into the category of regulation of transcription, suggesting that MXD3 may be part of a transcriptional cascade. The complete results are presented in **Table S2**.

## Discussion

Several lines of evidence suggest that MXD3 is an atypical member of the MAD family of transcription factors. MXD3 is transiently upregulated during GNP proliferation in the cerebellum in a pattern similar to that of Cyclin D2 [14]. MXD3 has been shown to be necessary and sufficient for SHH-mediated GNP proliferation [15], suggesting a role in the regulation of cell proliferation rather than differentiation or quiescence as demonstrated for MAD proteins. Recently, MXD3 has been shown to negatively regulate B cell differentiation in the spleen [16]. Besides MXD3 proliferative activity in normal development [14], [15], several pieces of evidence have emerged from disease models that point in the same direction. In *weaver* mice, where cerebellar GNPs fail to exit the cell cycle and differentiate [31], MXD3 fails to undergo the physiological downregulation observed in wild type littermates [14]. *Ptc^+/−^* mice, a model for medulloblastoma, show high expression of MXD3 in the tumor tissue but not in the adjacent normal postmitotic cerebellar tissue [15]. Database analysis suggests that *MXD3* is overexpressed in human cancers, predominantly in medulloblastomas and glioblastomas [18]. Taken together, these findings prompted us to explore the role of MXD3 in human medulloblastomas. Probing a small cohort of tumor samples by qRT-PCR validated the database analysis, supporting our initial hypothesis that MXD3 might be relevant to medulloblastoma proliferation. The results presented in this report support the conclusion that MXD3 is required for proliferation of DAOY medulloblastoma cells; however, persistent and/or high expression of MXD3 ultimately leads to apoptosis and cell death.

Two main aims in this study were to (i) analyze the role of MXD3 in proliferation of tumor cells and (ii) identify potential target genes and patterns of gene expression induced by this transcription factor as a first approach to understand the molecular mechanisms by which MXD3 exerts its function. Our experimental model consisted of knock-down or overexpression of MXD3 in DAOY cells. Two different siRNAs against *MXD3* resulted in decreased proliferation, in agreement with its role in GNP proliferation during normal cerebellar development [15]. Surprisingly, overexpression of MXD3 resulted in reduced cell numbers, in apparent disagreement with the results discussed above and with previous reports by our group and others [15], [16]. These seemingly contradictory results likely represent distinct phases of MXD3 activity, namely proliferation followed by apoptosis. Furthermore, this might also reflect why MXD3 levels are so low in the medulloblastoma-derived DAOY cell line. Several possibilities might explain these findings. First, MXD3 might trigger a proliferative response which in turn triggers cellular homeostatic mechanisms to control proliferation. This possibility is in agreement with previous results from our laboratory which, in a normal cerebellar development model, reported MXD3 proliferative activity was followed by induction of apoptosis [15]. Remarkably, although most cells died after an initial burst of proliferation in MXD3-transfected GNPs, those that survived and continued to express MXD3 were found to be mitotic [15]. These data indicate that MXD3 direct transcriptional regulation could account for both phenotypes described in this paper, namely proliferation (upregulation of putative direct targets IRF4, FBL, HTR1B) and growth arrest and apoptosis (upregulation of RUNX1T1 and BMP3; downregulation of USP2 and MYCL1) as shown in **Table 1**. In this regard, MYC proliferative and apoptotic activities are well documented (for review, see [32]). Recently, ARF has been reported as a switch for c-MYC, such that during physiological low-ARF conditions c-MYC drives expression of canonical targets that result in proliferation, while high-ARF (induced by oncogenic stress) directly inhibits canonical c-MYC target expression and induces non-canonical targets leading to apoptosis [33]. Thus, it is conceivable that such a molecular switch exists for MXD3, representing a fail-safe mechanism to prevent MXD3 driven hyper-proliferation. In this sense, yet uncharacterized binding partners might act as switches that define the MXD3 mediated response.

The data presented here highlights several aspects of MXD3 atypical function, consistent with an emerging body of evidence regarding the MYC/MAD proteins. For example, MNT (a Mad family protein) has been found to bind DNA targets that are not shared by either MYC or MAX [34], suggesting it participates in alternative mechanisms of gene regulation. Likewise, MAX-independent functions of MYC have been reported [35]. Interestingly, sequence analysis indicates that most MXD3 binding sites at promoter regions lie within or very close to MYC-type E-boxes, suggesting the intriguing possibility that it may bind target genes as a heterodimer with MYC. Indeed, MXD3 is predicted to interact with MYC proteins to a similar extent as the known binding partner MAX [18]. As another example, MXD3 has recently been shown to bind directly to the Id2 promoter and induce (rather than repress) transcription of the gene by histone acetylation (rather than deacetylation) via recruitment of HATs (rather than HDACs) [16]. MXD3 binds the Id2 promoter at an E-box, which is typical for other bHLH proteins. However, it is not clear whether the atypical recruitment of HATs is mediated by the SID domain. In line with this report, the majority of MXD3 targets that we identified are upregulated, in contrast with the classical transcriptional repression activity of other family members.

To our knowledge, this is the first report on a genome-wide approach to identify direct MXD3 targets. Based on ChIP-chip experiments, we present a list of candidate genes that seem to be directly bound by MXD3 to modulate transcription in DAOY medulloblastoma cells. Our results provide a significant contribution for future experimentation aimed at elucidating the role of MXD3 in tumorigenesis as well as in normal development.

The classical switch model of the MYC/MAX/MXD network, in which competition between MYC-MAX and MXD-MAX heterodimers for shared DNA binding sites controls proliferation or differentiation, needs to be expanded to accommodate these emerging challenges. MXD3’s dual effect on medulloblastoma cell proliferation may be another example of this increasingly complex regulation.

## Materials and Methods

### Proliferation Assays

Proliferation of DAOY and MXD3 stable cell lines was assessed by plating DAOY cells (American Tissue Culture Collection) in 6-well plates at 1,500–3,000 cells/well. Medium was changed daily and cells counted for 10–15 days. Proliferation assays with MXD3 constructs or siRNAs were performed by transiently transfecting 10^5^ cells/well in 6-well plates; cells were counted 48 hrs after transfection. In all cases, attached cells were trypsinized and counted using a Coulter Counter Z1 instrument (Beckman Coulter). Two replicates were counted for each data point, in at least three independent experiments.

### RNA Isolation and Expression Profiling

Total RNA was extracted from DAOY or MXD3 stable cell lines (0.5–1×10^6^ cells) using the RNeasy kit (Qiagen). For extraction of RNA from tumors, tissue was first homogenized in Trizol (Invitrogen) and after chloroform extraction, RNA was purified with RNeasy kits. RNA quality was assessed with a Bioanalyzer instrument (Agilent).

For expression analysis, RNA was amplified and biotinylated using Illumina TotalPrep RNA amplification kit (Ambion). Hybridization to Illumina Human Ref-8 v2 Expression BeadChips (22,000 transcripts) was performed by the Expression Analysis Core at UC Davis.

### Chromatin Immunoprecipitation and DNA Promoter Arrays

Chromatin immunoprecipitation (ChIP) assays were performed following published protocols [36]. Briefly, 10^7^ cells per assay were fixed with 1% formaldehyde for 10 min at room temperature (RT), followed by addition of glycine (125 mM final concentration) for 5 min. Fixed cells were collected with a cell-scraper, washed once with phosphate buffered saline (PBS) and either used immediately or snap-frozen and stored at −80°C. Fixed cells were homogenized in cell lysis buffer (5 mM Pipes pH 8.0, 85 mM KCl, 1% NP40) and pelleted nuclei were lysed in nuclear lysis buffer (50 mM Tris pH 8.0, 10 mM EDTA, 1% SDS). DNA was fragmented to ∼200 bp and immunoprecipitated with mouse anti-HA (Covance, cat# MMS-101R) and secondary rabbit anti-mouse IgG (MP Biomedicals, cat# 55436) antibodies. Nonspecific rabbit IgG (Alpha Diagnostics, cat # 20009–5) was used as a negative control and mouse anti-RNA polymerase II 8WG16 IgG2a (Covance, cat # MMS-126R) as a positive control. Cross-links were reversed in eluted immunoprecipitates and DNA was purified using QIAquick PCR purification kit (Qiagen). Precipitated DNA from ChIP with smaller starting material (4–30 µg/assay) was measured by Quant-iT PicoGreen assay (Invitrogen). Amplicons were prepared as previously described using the GenomePlex WGA4 kit (Sigma). DNA fragments were labeled by Klenow extension using 9-mer Cy3-primers for total (unenriched) input and Cy5-primers for immunoprecipitated material. Briefly, 1 µg of DNA and primers (1 O.D./42 µl) were denatured at 98°C for 10 min and incubated with Klenow (50 U/µl) and 1 mM dNTP at 37°C for 2 hrs. After stopping the reaction with 50 mM EDTA, DNA was precipitated with isopropanol and resuspended in H_2_O. Thirteen µg of the test and reference samples were combined, vacuum-dried, resuspended in array hybridization buffer (Nimblegen) and hybridized to Human HG18 RefSeq 385K promoter arrays (Nimblegen) for 16–20 hrs according to manufacturer’s instructions in a MAUI station (BioMicro) at 42°C.

### Cell Cycle and Apoptosis

Cell subpopulations at the different phases of the cell cycle were quantified by DNA content measured by flow cytometry. Briefly, 10^6^–10^7^ cells per sample were trypsinized, washed with PBS, resuspended in 0.5 ml of PBS and added to 4.5 ml 70% ethanol on ice. After 2 hrs, the fixative was removed, the cells were washed with PBS and resuspended in 1 ml of 20 µg/ml propidium iodide, 0.1% Triton X-100, 200 µg/ml RNase A in PBS. After 30 min at RT, data (50,000 events/sample) were acquired in a Becton-Dickinson FacScan instrument and fitted with FlowJo software (Tree Star, Inc.) using the Watson Pragmatic model.

For apoptosis analysis, cells were gently detached with 0.5 mM EDTA/PBS for 30 min at 37°C, washed with Hank’s Buffered Salt Solution (HBSS; Invitrogen) and resuspended in the same medium at 1.5×10^6^ cells/ml. One ml of the cell suspension was stained with Vybrant DyeCycle/Sytox AADvance kit (Invitrogen) according to the manufacturer’s instructions. Data (10,000 events/sample) were acquired in a Becton-Dickinson LSRII instrument and analyzed with FlowJo software.

### DNA Constructs and siRNA

Full-length human *MXD3* coding sequence (including stop codon) was amplified by RT-PCR from total RNA extracted from DAOY cells and ligated into pHM6 (Roche) to create an N-terminal-HA tagged expression vector. Single amino acid substitutions (E66K and E66D) were generated in the same vector backbone using a QuikChangeII Site-Directed Mutagenesis Kit (Stratagene) and the mutagenic primers 5′-GTG CAC AAT GAA CTG AAG AAG CGC AGG AGG G-3′, 5′-CCC TCC TGC GCT TCT TCA GTT CAT TGT GCA C (E66K) and 5′-GCA CAA TGA ACT GGA TAA GCG CAG GAG GGC C-3′, 5′-GGC CCT CCT GCG CTT ATC CAG TTC ATT GTG C-3′ (E66D). Deletion constructs were generated from the pHM6-*MXD3* template by PCR using the following primers: ΔSID, 5′-CCG GGT ACC GAA CCC TTG GCC AGC AAC GAG CAT GGT TAT GCG TCC CT-3′ and 5′-CCG GAA TTC TCA TAG CCA GGC GCC GCC GC-3′; Δbasic, 5′-GCG CAG GAC AGC GGG GCC CAG TTG AAG CGG-3′, 5′-CCG CTT CAA CTG GGC CCC GCT GTC CTG CGC-3′, 5′-CCG GGT ACC GAA CCC TTG GCC AGC AAC AT-3′ and 5- CCG GAA TTC TCA TAG CCA GGC GCC GCC GC-3′; ΔC, 5′-CCG GGT ACC GAA CCC TTG GCC AGC AAC AT-3′ and 5′-CCG GAA TTC TCA GAC GAA GCC CCG CAG CAG CT-3′. siRNAs were from Ambion; sequences are as follows: siRNA1 (Ambion #122441) 5′-AUG GAC UAA AAG GAC CCU Utt-3′; siRNA2 (Ambion#122442) 5′-AGG ACC CUU GUG UGG GAA Ctt-3′; siRNAc (Ambion #AM4611) 5′-AGU ACU GCU UAC GAU ACG GTT-3′.

### Cell Culture and Transfection

DAOY cells were grown in MEM medium supplemented with 10% fetal bovine serum (FBS), 1 mM sodium pyruvate and penicillin/streptomycin. For transient expression, cells were transfected at 90–95% confluence using FuGene HD (Roche) at a 5∶2 ratio (µl reagent:µg DNA) or Lipofectamine 2000 (Invitrogen) at a 3∶1 ratio, according to manufacturer’s instructions. Two µg of DNA were used per well in 6-well plates. siRNA transfections were carried out using 2.5 µl Lipofectamine-2000 (Invitrogen) and 100 pmol siRNA per well in 6-well plates with cells at 80–90% confluency.

For the generation of stable lines, cells were transfected as described above in 100 mm dishes. After 48 hrs, G418 (Cellgro) was added to a final concentration of 800 µg/ml and colonies were picked and expanded after 2–3 weeks. All stable cell lines used in the experiments described here were maintained in culture for more than 40 passages with no obvious change in phenotype.

### Quantitative Real Time PCR (qRT-PCR)

Reverse transcription reactions were performed using the High Capacity RNA to cDNA Kit (Applied Biosystems). RNA from tumors and stable cell lines was obtained as described above. Frozen tumor specimens were obtained from the Human Cooperative Tissue Network. Normal human developing and mature cerebellum RNA was purchased from BioChain. Quantification of *MXD3* mRNA was performed using the StepOne Plus Real-Time PCR System (Applied Biosystems). Validated assays for human *MXD3* and endogenous control 18S ribosomal RNA were used (Applied Biosystems). Experiments were performed in triplicate for each data point. Relative expression of *MXD3* mRNA level was calculated by the comparative C_T_ method.

### Other Methods

Immunoblotting was performed by separating protein extracts on 12 or 15% polyacrylamide gels under denaturing and reducing conditions. Gels were transferred to nitrocellulose and blots probed as described [15]. All primary antibody incubations were performed at 4°C overnight at the following dilutions: anti-HA, 1∶3,000; anti-tubulin, 1∶30,000; anti-GAPDH, 1∶1,000; anti-lamin, 1∶1,000.

Immunofluorescence was performed essentially as described [15]. Paraformaldehyde-fixed cells were permeabilized, blocked and incubated with anti-HA (1∶1000) at 4°C overnight.

Cellular fractionation was performed by incubating cells in cell lysis buffer for 20 min at 4°C followed by homogenization in a Potter-Elvehjem homogenizer. After centrifugation, the supernatant was reserved as the cytoplasmic fraction; the pellet was washed and pelleted nuclei were lysed in nuclear lysis buffer (50 mM Tris pH = 8.0, 10 mM EDTA, 1% SDS).

Protein concentration was determined using a micro BCA protein assay kit (Pierce).

Transcription factor binding sites were predicted using PROMO (http://alggen.lsi.upc.es/cgi-bin/promo_v3/promo/promoinit.cgi?dirDB=TF_8.3), a transcription factor analysis tool using version 8.3 of the TRANSFAC database (http://www.gene-regulation.com/pub/databases.html). The following transcription factor accession numbers were used: MYC: T00140 (consensus CACGTG), T00142 (consensus GGCCACGTGACC) and T00143 (consensus CACGTGG); MAX: T05056 (consensus ACACGTGGTAT) and T00489 (consensus CAGACCACGTGGTC); MYCN: T01445 (consensus CACGTG).

Statistical analysis was performed with Prism 5 (Graphpad Prism Software, Inc).

## Supporting Information

Figure S1 **Characterization of stable cell lines.** DAOY cells stably transfected with HA-*MXD3* (M1–M6) or with the empty vector (C1–C3) were tested for MXD3 expression by real-time PCR, immunoblot and immunofluorescence. **(A)** *MXD3* mRNA levels in six representative “M” lines and three representative “C” lines determined by quantitative RT-PCR analysis. Values represent the fold-increase in mRNA (mean of n = 4± SD), normalized to the normal mature cerebellum sample for comparison with figure 1. Note the logarithmic scale of the Y-axis. All “M” lines showed levels of *MXD3* message significantly higher than those observed in either normal tissue or the parental DAOY cell line (*p<0.001, n = 3, t-test). None of the “C” lines showed significant expression of the transgene above that of the parental cell line. **(B)** Analysis of protein levels in four representative “M” lines and the control line C1. Protein levels (anti-HA immunoblots) correlate with message levels in (A). HA-MXD3 was absent in the representative control line and was only detected in nuclear fractions of the M lines, but was absent from cytoplasmic fractions. Cytoplasmic and nuclear extracts were confirmed by detection of GAPDH and lamin respectively; these markers were also used as load controls. Anti-lamin antibody detects a double band; anti-GAPDH antibody detects a non-specific band of lower molecular weight in the nuclear fraction in addition to a specific band in the cytoplasmic fraction; the purity of the cytoplasmic fraction was further confirmed with anti-tubulin antibodies (not shown). **(C)** Nuclear localization of HA-MXD3 was also confirmed by immunostaining with anti-HA antibody (red) and counterstaining with DAPI for nuclei (blue). Images correspond to M1 and C1 cell lines and are representative of all other lines within each group. Scale bar, 100 µm.
(TIF)

Figure S2 **Mutagenized MXD3 expression constructs.** Schematic representation of the MXD3 expression constructs used in this analysis. Nomenclature is as follows: ΔSID, deletion of the SID domain; Δbasic, deletion of the basic region of the bHLHZ domain; ΔC, deletion of the 14 C-terminal residues; E66D, E66N and E66Q, single amino acid substitutions of the glutamic acid residue at position 66 of the basic region. Residue positions are indicated above; numbering corresponds to the human MXD3 protein RefSeq sequence (accession number NP_112590). Calculated relative molecular masses (in KDa) are indicated on the right.
(TIF)

Figure S3 **Mutagenized MXD3 protein localizes to the nucleus.** All MXD3 mutant proteins localized to the nucleus. Confocal images of DAOY cells transfected as indicated on the left; immunodetection of the expressed protein was performed with anti-HA (first column, blue) and anti-MXD3 (green). As evidenced in merged images, HA-MXD3 and mutated forms localized exclusively to the nucleus. Scale bar, 10 µm.
(TIF)

Table S1 **MXD3 target genes and expression profiles.** *Genes in this list were selected from ChIP-chip results based on the following criteria: peak values >3 in at least one biological replicate and/or peak values >2 in both biological replicates. Mxd3 targets that were validated by PCR with an independent ChIP sample are highlighted in yellow. ¶ChIP 1 and ChIP 2 represent the peak values of two biologically independent experiments and are calculated from promoter array signals using the Max4 algorithm. Significant peak values are highlighted in blue (>3) or light blue (>2). &Expression profile values represent fold increase in mRNA abundance calculated as in Table 1. Based on the criteria described in the Results section, upregulated genes are highlighted in red and downregulated genes are highlighted in green. N/A indicates genes not present on the array platform used.
(XLS)

Table S2 **Gene Ontology analysis of MXD3 target genes.**
(XLS)
