# Supplementary figures and images for: Role of MXD3 in Proliferation of DAOY Human Medulloblastoma Cells
Source: PLoS One. 2012 Jul 10;7(7):e38508. doi: 10.1371/journal.pone.0038508 (PMC3393725; doi:10.1371/journal.pone.0038508)

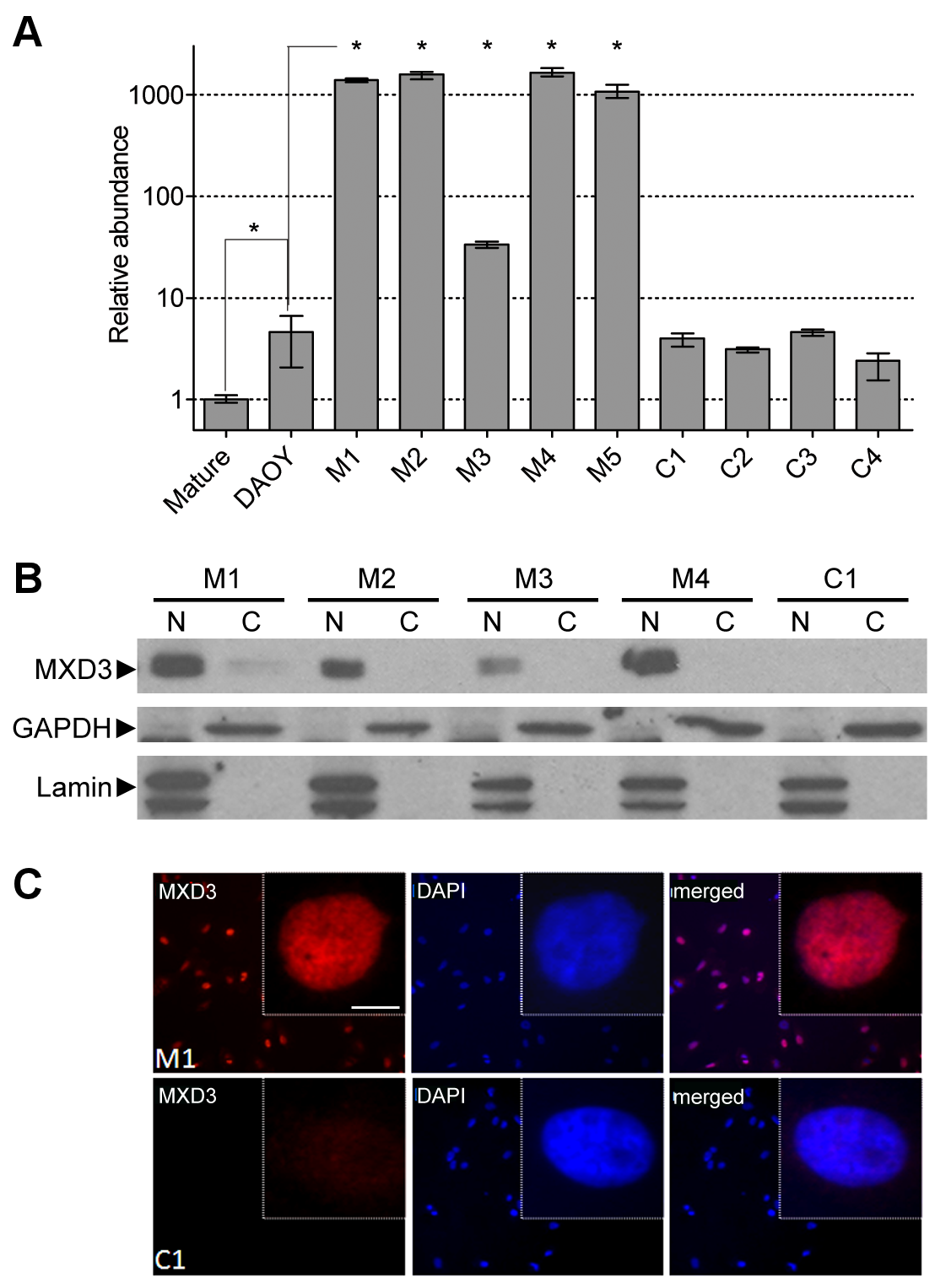

Supplement: Figure S1 — Characterization of stable cell lines. DAOY cells stably transfected with HA-MXD3 (M1–M6) or with the empty vector (C1–C3) were tested for MXD3 expression by real-time PCR, immunoblot and immunofluorescence. (A) MXD3 mRNA levels in six representative “M” lines and three representative “C” lines determined by quantitative RT-PCR analysis. Values represent the fold-increase in mRNA (mean of n = 4± SD), normalized to the normal mature cerebellum sample for comparison with figure 1. Note the logarithmic scale of the Y-axis. All “M” lines showed levels of MXD3 message significantly higher than those observed in either normal tissue or the parental DAOY cell line (*p<0.001, n = 3, t-test). None of the “C” lines showed significant expression of the transgene above that of the parental cell line. (B) Analysis of protein levels in four representative “M” lines and the control line C1. Protein levels (anti-HA immunoblots) correlate with message levels in (A). HA-MXD3 was absent in the representative control line and was only detected in nuclear fractions of the M lines, but was absent from cytoplasmic fractions. Cytoplasmic and nuclear extracts were confirmed by detection of GAPDH and lamin respectively; these markers were also used as load controls. Anti-lamin antibody detects a double band; anti-GAPDH antibody detects a non-specific band of lower molecular weight in the nuclear fraction in addition to a specific band in the cytoplasmic fraction; the purity of the cytoplasmic fraction was further confirmed with anti-tubulin antibodies (not shown). (C) Nuclear localization of HA-MXD3 was also confirmed by immunostaining with anti-HA antibody (red) and counterstaining with DAPI for nuclei (blue). Images correspond to M1 and C1 cell lines and are representative of all other lines within each group. Scale bar, 100 µm. (TIF) [file pone.0038508.s001.tif]

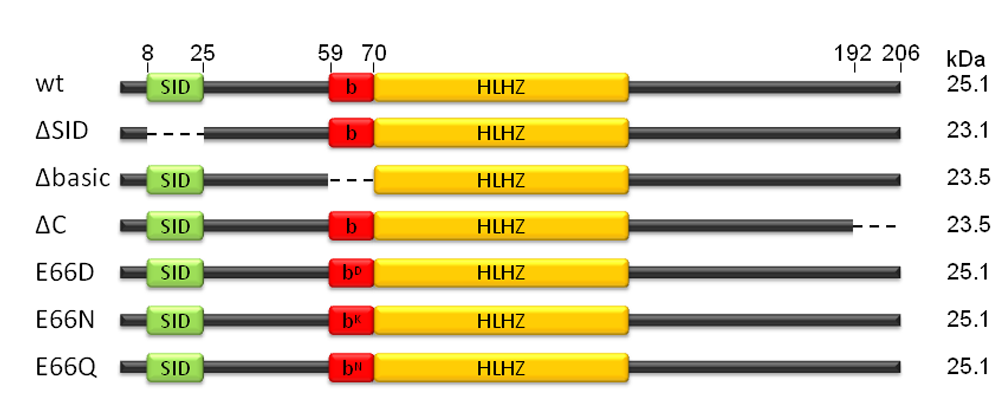

Supplement: Figure S2 — Mutagenized MXD3 expression constructs. Schematic representation of the MXD3 expression constructs used in this analysis. Nomenclature is as follows: ΔSID, deletion of the SID domain; Δbasic, deletion of the basic region of the bHLHZ domain; ΔC, deletion of the 14 C-terminal residues; E66D, E66N and E66Q, single amino acid substitutions of the glutamic acid residue at position 66 of the basic region. Residue positions are indicated above; numbering corresponds to the human MXD3 protein RefSeq sequence (accession number NP_112590). Calculated relative molecular masses (in KDa) are indicated on the right. (TIF) [file pone.0038508.s002.tif]

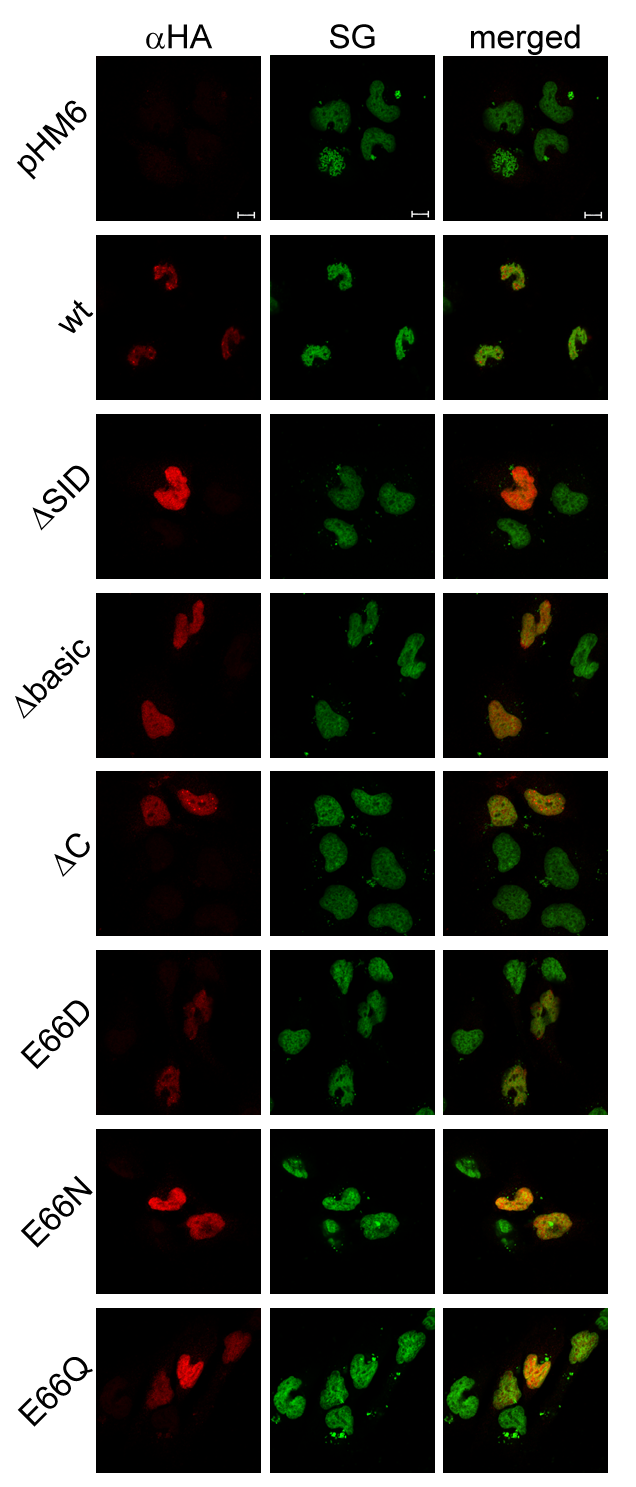

Supplement: Figure S3 — Mutagenized MXD3 protein localizes to the nucleus. All MXD3 mutant proteins localized to the nucleus. Confocal images of DAOY cells transfected as indicated on the left; immunodetection of the expressed protein was performed with anti-HA (first column, blue) and anti-MXD3 (green). As evidenced in merged images, HA-MXD3 and mutated forms localized exclusively to the nucleus. Scale bar, 10 µm. (TIF) [file pone.0038508.s003.tif]
